# “People just don't understand their role in it.” Collaboration and coordination of care for service users with complex and severe mental health problems

**DOI:** 10.1111/ppc.12633

**Published:** 2020-10-08

**Authors:** Eva Biringer, Oddbjørn Hove, Øivind Johnsen, Haldis Økland Lier

**Affiliations:** ^1^ Section of Research and Innovation Helse Fonna HF Stord Norway; ^2^ Stord Community Mental Health Center Helse Fonna HF Stord Norway; ^3^ Helse Fonna HF Haugesund Norway

**Keywords:** collaboration, continuity of care, coordination, mental health care

## Abstract

**Purpose:**

To explore professionals' and service users' experiences and perceptions of interprofessional collaboration and coordination for service users with complex and severe mental health issues.

**Design and Methods:**

A qualitative study involving semi‐structured interviews of professionals and individual interviews of service users. Data were analyzed by thematic analysis.

**Findings:**

Participants described challenges and suggested improvements concerning *Distribution of roles, responsibilities, and tasks; Communication; and Knowledge and attitudes*.

**Practice Implications:**

Mental health nurses and other professional helpers should have a particular focus on common aims, clear division of roles, planning and timing of interventions, and communication with other professionals and service users.

## 1 INTRODUCTION

Collaboration and coordination of care are associated with service user satisfaction and improved outcomes.^1^, ^2^ Interprofessional *collaboration* involves different health and social care professions, such as mental health nurses or social workers in community mental health services or specialist mental health services, that regularly work together to solve problems and provide services. Interprofessional *collaboration* can be defined as “both a process and an outcome in which shared interest or conflict that cannot be addressed by any single individual is addressed by key stakeholders.”^3^, ^4^ Interprofessional *coordination* differs from interprofessional *collaboration*, as it is a “looser” form of working arrangement, whereby interprofessional communication and discussion are less frequent.^5^

Previous studies have shown that service users experience suboptimal coordination and collaboration in and among services that are relevant for recovery from mental health and addiction problems.^6^, ^7^, ^8^, ^9^, ^10^ Professionals and service users have pointed to insufficient planning and coordination, inconsistency of approaches, and frequent breaks in personal relations with service users.^9^, ^10^, ^11^, ^12^, ^13^, ^14^, ^15^, ^16^, ^17^ Such breaks reduce relational and information continuity and imply a risk to patient safety.

Some service users with complex and severe mental health problems are experienced as particularly challenging to relate to and provide appropriate care for by nurses and other professional helpers. These service users need coordinated, continuous care from a range of practitioners in multiple health and social care services. They often have cooccuring substance abuse, are overrepresented in police interactions and compulsory treatment^18^, ^19^ and account for a majority of spendings in health and social care.^20^ Mental health nurses and other professional groups spend much of their time and resources on this group of service users. Optimal collaboration and coordination for and with this group are particularly important, as it is a group in need of continuous and consistent care and it is particularly vulnerable to breaks in relational and informational continuity.^21^ However, there is a gap in the evidence pertaining to collaboration and coordination with and for these service users with complex and severe mental health issues.

### 1.1 Aim

This multiperspective study aimed to explore the collaboration and coordination among service providers relevant for service users' recovery. Further, the study aimed to inform future improvement of interprofessional collaboration and coordination by exploring professionals' and service users' perceptions and opinions of how collaboration and coordination could be improved. Multiple perspectives, including the service user perspective, are relevant when exploring collaboration and coordination in and among services for service users with complex and severe mental health issues. The following research questions were asked: How do service users and professionals experience and perceive interprofessional collaboration and coordination? How—in their opinion—could collaboration and coordination for service users with complex and severe mental health problems be improved?

## 2 METHODS

### 2.1 Study design and setting

The study was a qualitative multiperspective study conducted in the geographical area of a Local Health Trust in Norway. In Norway, the responsibility of providing primary health services lies with the communities and specialist mental health and addiction services lie with the health trusts. In the communities, community mental health nurses, mental health community teams, supported housing, day centers, and home‐based services are available. General practitioners (GPs) and primary care emergency services in the communities serve as gatekeepers to specialist services. The police are involved in bringing in service users considered a risk to themselves or others to their GP or the primary care emergency center. GPs there may further refer service users to community mental health centers (CMHCs; secondary care) or to hospitals (tertiary care) for specialist outpatient or inpatient treatment. Outreach teams at the CMHCs are responsible for service users with complex and severe mental health and/or addiction issues who are particularly hard to reach or challenging to relate to. Further relevant institutions are child protection services and the Norwegian Labour and Welfare Administration (“NAV”). Each service has an electronic journal system with information that is only available to the specific service. Access to information is strictly regulated by legislation.

### 2.2 Participants

Six individual interviews with service users with complex and severe mental health problems were performed. They were all using the outreach team at a CMHC responsible for mental health specialist services to a population of approximately 34,000. The outreach team served 40 service users with severe mental health conditions, very difficult life situations, and behaviors that were perceived by professionals as particularly challenging. The service users were invited to participate in the study via their primary contact at the outreach team. They were interviewed about their experiences as service users. The semi‐structured interview guide included questions about collaboration and coordination in terms of how the service users experienced that the professionals understood and responded to their needs, how professionals worked together, and how collaboration and coordination could be improved. Five interviews were conducted in the participant's home and one in a car at a parking lot.

Nine homogeneous group interviews with mental health nurses and other professional helpers from relevant services were conducted. The semi‐structured interview guide included questions about how participants experienced and perceived collaboration and coordination for service users with complex and severe mental health problems, and how collaboration and coordination for these service users could be improved. To increase the internal validity of the study, professionals were asked to describe service users they perceived as particularly challenging to relate to and provide appropriate care for before they were asked about their experiences and perceptions of collaboration and coordination for and with these service users.

Interviews lasted approximately one to one and a half hours; they were audiotaped and transcribed verbatim.

### 2.3 Analysis

A data‐driven stepwise procedure in line with thematic analysis was used.^22^ The data analysis proceeded as follows: the first author systematically coded all text material and defined preliminary themes. The researchers discussed and agreed upon a common understanding of the semantic and latent constructs underlying the material in the preliminary themes. Based on the common understanding reached during these discussions, the first author made the final categorization of the contents and drafted the manuscript based on the notes from the discussions. To ensure the internal validity of the study, results were compared with the original transcripts throughout the writing process. Acknowledging that the researchers' own involvement and prior understanding may influence on which knowledge is acquired,^23^ reflexivity was practiced throughout the study by holding team meetings and discussing possible interpretations of semantic contents and latent constructs underlying the data. Coding was performed in N'Vivo 12.

### 2.4 Ethics

Approval for this study was obtained from the Norwegian Social Science Data Service (ref. no.: 2013‐32446) and the Regional Committee for Medical Research Ethics (ref. no.: 2011‐2262). Participants were informed about the voluntary nature of their participation, their right to decline without any negative consequences and confidentiality. All of the participants provided written informed consent to participate. Interview transcripts and audio recordings are stored securely according to national legal requirements.

## 3 RESULTS

The characteristics of public services, professionals, and service users are shown in Table 1. Out of the 36 participants with professional backgrounds, 28 were women and 14 were nurses. All participating service users were male and their age ranged from 21 to 69 years; the mean age was 37 years. All service users had severe problems in a range of areas—including family, social network, work, and housing—and all of them depended on social benefit payments for their daily support.

**Table 1:** Public services and participating professionals (*N* = 36) and service users (*N* = 6)

Service	Service function	Background^a^	Gender
**Professionals**			
In‐hospital emergency communication center “Akuttmedisinsk kommunikasjonssentral (AMK)”	Handles somatic and psychiatric emergency communication, i.e., emergency phone calls, referrals to specialist services, and coordination of patient transport	Nurse: 4	M: 0 F: 4
Primary care emergency center “Legevakt”	Out‐of‐hours service with on‐call GPs who refer service users to specialist care	GP: 3	M: 1 F: 2
Community mental health services “Psykiatritjenesten”	Provide ongoing follow‐up of service users including home‐based supervision on household maintenance and personal care	Mental health nurse/leader: 1 Mental health nurse: 4	M: 0 F: 5
Community home care services “Hjemmebaserte tjenester”	General home nursing services available 24/7	Leader: 1 Nurse: 1 Nurse/environmental therapist: 4	M: 0 F: 6
Acute psychiatric ambulatory team in specialist mental health services “Akuttambulant team (AAT)”	Provides emergency specialist evaluation of service users in crisis after referral by the on‐call doctor at the emergency primary care emergency center, a GP or the police. Advisory function for on‐call GPs and employees in the community mental health services	Psychologist: 3	M: 2 F: 1
On‐call physicians specializing in psychiatry at the psychiatric emergency departments “Akuttvakt”	Responsible for evaluation and initial treatment of acutely referred service users	Psychiatrist: 2 Physician: 2	M: 2 F: 2
Police	Patrolling units that bring in service users at risk of harming themselves or others for evaluation at the primary care emergency center before emergency admission to specialist mental health care	Leader: 1 operative leader: 1 Police officer: 2	M: 3 F: 1
Labour and Welfare Administration “NAV”	Responsible for pensions, unemployment and sickness benefits, qualification programs, and employment schemes. Offers temporary financial assistance, temporal accommodation, financial advice, and debt counseling	Consultant/advisor: 3	M: 0 F: 3
Child protection services “Barnevernet”	Responsible for prevention, follow‐up, and support of children at risk and their families. Provides supervision, various types of help and support, respite care or relocation of children	Consultant: 4	M: 0 F: 4
Service users	Using the outreach team at the Community Mental Health Center. The team includes mental health nurses and psychologists offering frequent home visits, assistance with daily domestic tasks, and counseling with regard to practical, financial, and mental health and addiction issues. The team is also responsible for clinical evaluation and treatment	Psychosis: 5 Alcohol or drug abuse: 4 Custody: 2	M: 6

Abbreviations: F, female; GP, general practitioner; M, male.

^a^ Numbers in bottom row do not add up as service users had multiple problems.

**Figure 1:**
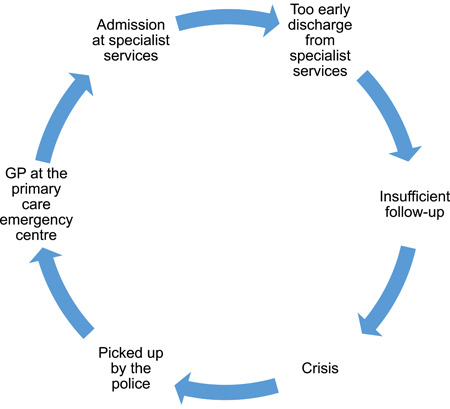
The “vicious circle” [Color figure can be viewed at wileyonlinelibrary.com]

Professionals provided rich and detailed descriptions of their experiences of collaboration and coordination in and among services as well as of personal and contextual characteristics of service users with complex and severe mental health problems who they perceived as challenging to relate to and provide appropriate care for. They described the situation of service users and their professional helpers as a “vicious circle” (Figure 1). The vicious circle started when the service users were discharged from or left specialist treatment. When the next crisis occurred—for example, their psychotic symptoms worsened, their use of drugs escalated or their next‐of‐kin became exhausted—the service users visited their GP or the primary care emergency center, or they were picked up by the police, to be admitted to specialist services again. Professionals' responses to service users' crises were described as “*back and fort*” and “*firefighting*,” with frequent disruptions in information flow and personal relationships between service users and professional helpers. Further, professionals experienced frustration because the tools or services that could have met the service users' needs sometimes were unavailable or did not exist, and as professionals frequently had to take on the unpleasant gatekeeper role when service users were supposed to get help via other services. In response to the question of what characterized service users they experienced as challenging to relate to and provide appropriate care for, professionals gave the following descriptions: chaotic lifestyle, threatening to take their own life, frightening or threatening others, do not wish to use services, do not keep agreements, disagree with professionals' views of problems, at the limit of their ability to take care of themselves, in need of care and support for many years, and lacking material resources. In terms of diagnoses, comorbid mental health and addiction problems, relapse of psychosis, unstable personality disorder, comorbid somatic conditions, and “diagnoses that professionals know little about” were mentioned.

**Figure 2:**
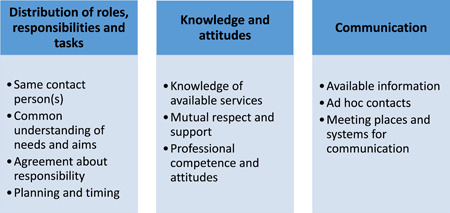
Themes [Color figure can be viewed at wileyonlinelibrary.com]

Three main themes reflecting health professionals' and service users' experiences, perceptions, and opinions of interprofessional collaboration and coordination were developed: *Distribution of roles, responsibilities, and tasks; Communication*; and *Knowledge and attitudes* (Figure 2).

### 3.1 Distribution of roles, responsibilities, and tasks

#### 3.1.1 Same contact person(s) and predictability

According to professionals and service users, stable predictable relationships between the service users and their helper(s) were important. Frequent changes of primary contact persons “*did not work”*:

That I have a regular treatment is the most important for me []. The regular contact keeps me stable. […]. So it's the continuity: that it's predictable and it's stable [that is important]. (P2, service user)

According to the service user, things easily “*slipped back to the old ways, [and] I fell to the side‐lines again”* when he experienced disruptions in the regular contact with his primary helpers. Several professionals emphasized early contact with the service users as crucial when providing treatment and support, as early contact helped professionals “get the service user on side.” Contact overtime was central to observe and evaluate the service users' situation and needs, and to “catch him the day he begins to fall away.”

Service users further emphasized the importance of being in control of their own situation and the things done to help them:

It has to be that way now that I'm in control, otherwise it'll be totally… Then I can't be bothered, if somebody tries to control how I am, what I should think is important and such. That's not ok for me. (P3)

#### 3.1.2 Common understanding of service users' needs and aims for treatment

From the point of view of professionals, prerequisites for the system to work optimally were each stakeholder's awareness of the goals of treatment and support as well as the role and contribution of each stakeholder. However, several participants described a messy situation around the service users in which there obviously were no common agreement about goals among professionals and no common plan for treatment and support:

All the things we think about in connection with them [the service users]: Trying to contact the GP, establish a relationship. Then they [the service users] get a diagnosis, then they [professionals] retract the diagnosis, then they [the service users] get a new one []… And it's forward and back … And we have to follow them round and round in circles … (P34, Community home care services)

The messy situation described above lead to frustration among all involved parties, and a lot of time and resources were spent with little results.

#### 3.1.3 Planning and timing of treatment and support

Several service users, for instance this service user with schizophrenia and a history of drug abuse, spoke of how they sometimes experienced the contact with professional helpers as unpredictable and out of their control:

And so it's been a bit unpredictable as sometimes there are weeks between each time we see each other, and then I suddenly get a message about it right before the meeting. I like having a bit of control and finishing things. (P5, service user)

Appropriate planning in and across services helped professionals and service users involve relevant services and timing interventions appropriately. This reduced risks and use of resources in the long run.

#### 3.1.4 Agreement about the responsibility for the service users

Disagreements about who was in charge of treatment and support were described as a common source of frustration and conflict among professionals. One border of conflict was between the specialist mental health services and the police when service users in crisis were brought in by the police:

It is the police versus us. Should the person “rest” in an overnight cell or should he get a crisis bed [at the psychiatric emergency ward]? And then there's the primary care emergency center; there they talk about “where? What is right? What is right address for this person tonight?” I find discussions like that … It's so bloody difficult! (P1, Acute psychiatric ambulatory team)

The other main border of conflict was between community care and specialist mental health services:

I have to admit that I really feel frustrated about the assigning of responsibility between the first and second line services, because sometimes it seems to me [] that it's almost a lottery about who gets the responsibility [for the service user]. And often it lands on the police, when… when nobody else takes responsibility, then it's us that have to sort things out. Like people just don't understand their role in it. (P28, police)

In particular, compulsory admissions to specialist mental health services were experienced as a “*big problem*” by professionals, as they were so troublesome to administer for the GPs and other involved parties. The route into specialist mental health services via the GPs on call at the primary care emergency center was experienced as a time‐consuming “*detour*”:

I think the most frustrating is that I feel that the primary care emergency center is the wrong place for these patients. I feel that I am the wrong person, and I have to handle them and I am responsible for them. They [the service users] need someone to talk to, they need backup, and they need to be heard…. And there isn't time and space for that at the primary care emergency center. (P24, GP at the primary care emergency center)

Many participants, however, experienced specialist mental health services as unwilling to take in service users in crisis, although no satisfactory solutions for treatment and support of the service users were available in the community. Professionals in specialist mental health services, in contrast, felt they were under pressure due to limited capacity in their services. One consequence of the limited capacity was premature discharges and the “vicious circle” described in Figure 1. Availability of services rather than support needs often determined which service the service users were offered. For instance, nurses in the community home care services frequently had extensive contact with severely ill service users because the nurses were available 24/7.

### 3.2 Communication

#### 3.2.1 Available information about the service users

Participants from the police, acute psychiatric ambulatory team, and GPs described that they sometimes had to “*jump into*” very challenging and risky situations without having the information necessary to deal with the service users in a professional, proper, and consistent way:

It is on the occasions where the danger level is not obvious that we fall short. Where health services most probably have useful information in relation to the [situational] assessment, but which is technically confidential. But which we don't agree is confidential. If it's that type, if the information is of such a nature that they [the service users] could threaten the life and health of the servicemen involved in the callout, then we believe to the contrary that health services have a duty to inform us. (P28, police)

Restrictions concerning the sharing of information and communication about the service users across services were frequently mentioned as causes for loss of information, delay in handling issues, extra hassles, and increased risk. Nurses in the community services responsible for following up with service users after discharge from the specialist mental health services were unsure of what to do and how to react when the service users' problems escalated, as they frequently did not have access to updated treatment‐ and crisis plans. Access to updated information about the service users' needs, their professional and social network, current situation, and updated plans for treatment was deemed necessary to make appropriate decisions.

#### 3.2.2 Ad hoc contacts

Accessibility and flexibility of services were important to service users, as these lead to quicker professional responses to their needs for treatment and support. For instance, several service users valued the opportunity of contacting the outreach team by phone when needed:

Then I have like an open line in case something happens. I can often just send a text message, then things get fixed quickly. (P5, service user)

Ad hoc contacts paved the way for more flexible solutions tailored to the need of the service users. Professionals valued ad hoc contacts with other professionals for exchange of advice and information. Such exhange made professionals feel that the decisions they made were appropriate and risks reduced.

#### 3.2.3 Meeting places and systems for communication

Professionals described the existence of “*brick walls*” between services. They complained that opening hours of most services were limited and reaching professionals by phone was difficult. They pointed to the need for effective systems for rapid exchange of information between services.

**Figure 3:**
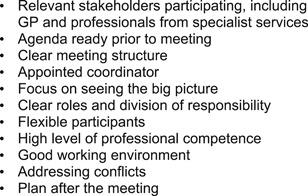
Success factors for well‐functioning support groups

Some service users had established interprofessional support groups that regularly met, with or without the service user present. Several service users found the group meetings useful, as they experienced that professional group members agreed on the measures to be taken and as the personal goals of the service users were central: “*We talk about what is important. I notice that they [the professionals] agree with what is important to me*.” Also, professionals experienced these group meetings as useful, although arranging them was “*an awful lot of paperwork and logistics*.” According to the participants, the group meetings resulted in fewer severe crises because of early and coordinated interventions by the group members, and the professionals were left feeling more secure that the way they dealt with challenging situations were meaningful. Success factors for well‐functioning interprofessional support groups according to participants are listed in Figure 3.

### 3.3 Professionals' knowledge and attitudes

According to participants, the professionals' background, training and experience constituted the basis for their views of service users' problems and how these should be handled. One of the consequences of the differing views among professionals and services was changes of diagnoses and alterations in treatment approaches:

If the patient is admitted to an institution where the therapist has an opinion about his condition, then he gets one type of treatment. And if, for example, the patient gets worse and needs to be transferred to the locked ward, there is another treatment with another viewpoint of the patient. Then suddenly the patient gets another type of treatment. And that can go on for a long time, … so in a way, nothing gets finished … And if that happens over many years… then the patient becomes a chronic case, that's my understanding. (P4, psychiatrist at an acute psychiatric ward)

Professionals further described a range of attitudes that they experienced as barriers to collaboration. They experienced lack of awareness of and respect for other services as well as cultural and language differences between professional groups. According to the participants, professionals not knowing enough about existing service alternatives, procedures and legislation caused delays, suboptimal treatment, and increased workload for the involved parties. For instance, the police accused professionals at the psychiatric wards of not having the necessary understanding of procedures and legislation in situations involving service users with potential harmful behavior. In their experience, varying interpretations of the statutory duty of confidentiality could be associated with increased risk. An incident in which a policeman was stabbed to death during such a callout was referred to as an example of this.

Positive relationships, mutual respect, and support among professionals in and among services were central for communication and coordination. However, as this participant from the Social Welfare Administration noted, several professionals experienced other stakeholders as distrustful or disrespectful:

Some doctors like to, you know… sit and deride the social welfare administration in such meetings, and I don't appreciate that, and it affects me. (P25, social welfare administration)

In particular, professionals in child protection service experienced distrust from others, which was hurtful and lead to further confusion about roles:

I experienced recently that a psychologist almost worked in opposition to the things I said, that he said something like “child protection services have carte blanche here, in relation to access to information”, and [he said to the service user]: “are you sure that you want to give a urine test? Because child protection services can use it against you”. Then I get really frustrated: “Who's side are we on now exactly? Are you on another side than me? I'm on the side of the child. Where are you?” (P13, child protection services)

Although this issue was not specifically addressed by participants, the transcripts revealed clear differences in language between groups of professionals, and between professionals and service users, when speaking about service users' conditions, needs, treatments and aims for treatment. This nurse pointed to the fact that language reflected basic differences in understandings and approaches between professional groups, and between professionals and service users:

Many of these [service users] have understandable symptomatology and understandable types of behaviour for each of the parts. But when it comes to translating from specialist health service level to municipal health service level, then the language usage is a little different. [] So people don't meet at the same level. So then the service user becomes a “challenging” service user. (P35, home care services)

Risk of miscommunications due to an insufficient mastery of the Norwegian language and limited knowledge about Norwegian cultural practices among some professionals coming from abroad were also mentioned several times.

I understand, in a way, that if you're a foreign doctor ‐and maybe has limited ability to catch nuances communicated in language‐, that treating a broken arm is easy, but maybe it's more difficult to treat this kind of illness [mental health conditions] if you [the doctor] can't “read between the lines” … (P28, police)

### 3.4 Suggestions for how to improve collaboration and coordination

Service users desired on‐going and predictable relationships with professional helpers, accessible and flexible help, possibility of ad hoc contacts, home visits and they wished to be in control of the situation. Professionals' suggestions for how to improve collaboration and coordination in and across mental health, social care and police services are shown in Table 2. Suggested improvements included the possibility of direct admissions of service users in crisis from primary care to specialist mental health services (see Figure 1), supervision from specialist mental health services, common communication systems for sharing updated treatment and support plans and improved possibility of ad hoc contact between service users and professionals and between services.

**Table 2:** Suggested improvements of collaboration and coordination

Suggested improvement	Source
Get to know the service user	Community mental health services
Use the necessary time to build a trusting relationship with the service user	Community mental health services
The environmental therapist services must not withdraw in phases in which the service user is stable	Community mental health services
Get involved with the service user as early as possible to avoid escalation of problems	Community mental health services Police
Diagnostic clarification is important, as it determines correct treatment and a lot of back‐and‐forth is avoided	Specialist mental health services
One senior psychiatrist should always be in charge for service users when involved professionals disagree about diagnosis and treatment	Specialist mental health services
Support with housing, work and treatment for mental health issues must be provided in parallel	Labour and Welfare Administration
A coordinator or primary contact should coordinate the help the service user gets	Labour and Welfare Administration Community home care services
The service user should have a plan for treatment and support	Community home care services
The service user should have an interprofessional support group	Labour and Welfare Administration
Mental health services must have a plan for regularly seeing service users who are “ticking bombs”	Police
The latest plans for treatment should be available to all involved parties	Acute ambulatory team Emergency communication center
Discharge summaries including plans and advice for how to deal with future crises should always be sent to the primary care emergency center	Primary care emergency center
Information about who is the primary health professional of the service user should be easily available	Labour and Welfare Administration
Updated information about scheduled appointments and the service users' professional network should be available	Primary care emergency center
The electronic patient record –system should automatically update the information regarding who the patient's GP is	Acute ambulatory team
There should be a communication system for quick and ad‐hoc information exchange across services	Labour and Welfare Administration
GPs should be experienced and available	Acute ambulatory team
There should be no distinction between specialist and primary care services	Primary care emergency center Community home care services
Direct admission to specialist mental health specialist care without having to visit the primary care emergency center first should be possible	Emergency communication center
Service users could have the possibility of direct contact with the acute ambulatory team (i.e., specialist services), without having to go to the primary care emergency center first	Primary care emergency center
The acute ambulatory team (i.e., specialist services) should more often and quicker come and see service users while they are at the primary care emergency center	Primary care emergency center
Mental health professionals must get out of their offices to evaluate service users who are “ticking bombs”	Police
Open low‐threshold 24/7 services in the communities offering acute accommodation, treatment, and support when the service user needs it should be established	Community home care services
The professional competence of the specialist mental health and addiction services should be more readily available for primary care services	Community home care services
Competence about mental health and addiction problems in community mental health services should be increased	Community home care services
A mental health nurse from the community mental health services should be available to the primary care emergency center	Primary care emergency center
Paper exercises help professionals get to know about available services and how and when these should ideally be contacted and work together	Acute ambulatory team
Child protection services and community mental health services should collaborate more about parents' mental health issues	Child protection services
Mental health services should focus more on the welfare of the children and more often send notifications of concern to the child protection services	Child protection services

Abbreviation: GP, general practitioner.

## 4 DISCUSSION

In this multiperspective study involving relevant services and service users, mental health nurses and other professionals pointed to mutual respect, common goals, information sharing, and knowledge of other available services as important aspects of collaboration and coordination. Information transfer shared understanding and working atmosphere are associated with handoff quality.^24^ As a comprehensive approach including a range of public services, including the police and child protection services, and first‐hand experiences were taken, this study most likely has high socio‐ecological validity and transferability.

Several participants used the term “brick walls” in their characterization of the current strict division between services and the division of health services into primary and specialist care. The possibility of direct admissions of service users in crisis from professionals in primary care to specialist mental health services, increased access to supervision from specialist mental health services in primary care, common communication systems for sharing updated treatment and support plans and improved possibility of ad hoc contacts between services were suggested as measures to improve collaboration and coordination. The quality of care and care transitions can be improved by bidirectional communication and planning,^25^ interventions incorporating continuing support during care transitions,^26^ and models of care with a high degree of interprofessional collaboration.^27^ Many international studies point to a gap between policy aspirations for care coordination and personalized care planning, and current practice.^28^ As for the organization of public mental health services, a central question raised by the present study was whether the same professionals should be responsible for a service user's care across inpatient and outpatient settings (continuity of care) or whether there should be separate teams (specialization).^29^ The current reforms in Europe are inconsistent with regards to the question of which to favor, although the current study and existing evidence suggest better outcomes and stakeholder preferences for continuity of care systems.^29^ In line with the present study, previous studies point to peer support and inclusion of family members in planning and follow‐up as central in collaboration and coordination.^26^, ^30^, ^31^ Integration of education of professionals and service user involvement in services may lead to mutual understanding and respect among professional groups and between service users and professionals. Fortunately, some recent developments have been made in Norway. Specialist addiction and mental health services are being colocated and electronic communication systems allowing for requests and transfer of administrative or clinical information between community care, GPs, and specialist services are available.

The study has some limitations that should be recognized. All researchers had professional backgrounds, and coresearchers with experience as service users were not included to strengthen the service user perspective in the study. Although the researchers were aware that their preconceptions could affect the questions asked and conclusions drawn, intersubjective elements may have influenced data collection and analysis. Information from service users was not very rich or in‐depth, and some experiences were schematically described. This may, however, suggest that the service users interviewed represented the intended group. The perspective of professionals in addiction services should have been included as most service users had substance abuse problems.

## 5 CONCLUSIONS

This multiperspective study provides a valuable comprehensive understanding of collaboration and coordination for and with service users in and across mental health and social care and the police. The study points to a need for involvement of service users and next‐of‐kin, interprofessional education, supervision, and meeting places and systems for communication to improve collaboration and coordination. Personal planning, care coordinators, and support groups are valuable tools for the improvement of relational continuity and coordinated assistance with housing and work, and mental health care. Professionals point to a gap between existing services and service users' needs. Structural and legal changes are still necessary to improve care for service users with severe and complex mental health problems.

### 5.1 Implications for nursing practice

The gap in collaboration and coordination between professionals and services is consistently identified as a major impediment needing effective solutions. Findings from this paper emphasize the value of nurses' and other professionals' efforts to improve communication with professionals, service users, and next‐of‐kin. Mutual respect, common goals, information sharing, and knowledge of other available services are important aspects of collaboration and coordination. Collaboration and coordination can significantly improve the quality of service delivery for persons with complex and severe mental health issues, that is, service users who are particularly vulnerable to breaks in the continuity of care.

## CONFLICT OF INTERESTS

The authors declare that there are no conflict of interests.
